# Factors determining exercise capacity in patients with atrial septal defect: assessment of heart function with CMR during dobutamine stress

**DOI:** 10.1186/1532-429X-17-S1-Q85

**Published:** 2015-02-03

**Authors:** Sigurdur S Stephensen, Katarina Steding-Ehrenborg, Ulf Thilén, Johan Holm, Peter Hochbergs, Håkan Arheden, Marcus Carlsson

**Affiliations:** 1 Department of Pediatric Cardiology, Lund University Hospital, Lund, Sweden; 2 Cardiac MR group Lund, Dept. of Clinical Physiology, Lund University, Lund, Sweden; 3 Department of Cardiology, Lund University Hospital, Lund, Sweden; 4 Dept. of Medical Imaging and Physiology, Lund University Hospital, Lund, Sweden

## Background

Patients with left to right shunting of blood through an atrial septal defect (ASD) have decreased exercise capacity. This study hypothesized that central factors influence exercise capacity, namely systemic and pulmonary cardiac output and right ventricular (RV) function during stress as well as left atrial pressure (LAP) and pulmonary artery pressure (PAP). Previous studies have found varying effects of stress and increased heart rate on the degree of shunting. The purpose of the study was therefore to determine if atrial shunting ratio changes during stress and examine if central factors can explain decreased exercise capacity in ASD patients.

## Methods

Eighteen patients with ASD and 16 healthy volunteers underwent cardiac magnetic resonance at rest and during 20 µg/kg/min dobutamine infusion and 0.25-0.75 mg atropine injection, aiming for an increase in heart rate to at least 70% of age-predicted maximal pulse. Two patients could not undergo stress CMR. Cine ssfp images were used for LV and RV volumes. Flow velocity mapping of the aorta and pulmonary trunk quantified cardiac output and shunt ratio (QP/QS) at rest and during stress. Ergospirometry was used to determine peak oxygen uptake (VO_2_*peak*). LAP and PAP were invasively measured at rest at the time of transcutaneous closure of the ASD.

## Results

**Figure 1:**
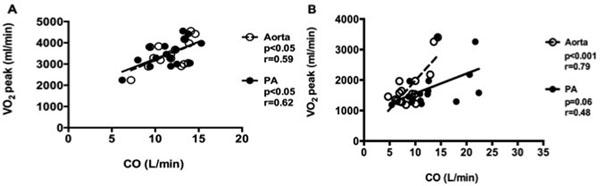
Linear regression analysis between systemic (Aorta, dotted line) and pulmonary (PA, solid line) cardiac output during dobutamine stress and VO_2_ peak in healthy controls (A) and patients with ASD (B).

Subject characteristics are shown in table 1. In patients with ASD the shunt ratio decreased from 2.2±0.8 at rest to 1.6±0.6 (p<0.01) during dobutamine stress. On dobutamine stress systemic cardiac output increased by 81±37% and pulmonary cardiac output increased by 30±28% (p<0.001). VO_2_*peak* correlated with cardiac output at dobutamine stress in controls (fig 1A), but only with aortic cardiac output in patients with ASD (fig 1B). VO_2_ peak did not correlate with QP/QS in patients (p=0.22 at rest and p=0.29 at stress). VO_2_ peak correlated with RV end systolic volume during stress in controls (p<0.01) but not in patients (p=0.56). There was no correlation between VO_2_ peak and LAP or PAP at rest (p=0.45 and p=0.71 respectively).

**Table 1:** Subject characteristics

	ASD patients		Healthy controls	
N	18		16	

Mean age and range	51±18		35±13	

Females, n (%)	13 (72)		3 (19)	

BSA, m2	1.9±0.2		1.9±0.2	

Peak oxygen uptake on ergospirometry (ml/min)	1795±661		3482±624	

	Rest	Dobutamine stress	Rest	Dobutamine stress

Heart rate, bpm	73±11	124±11	65±19	134±12

Cardiac output (L/min)	4.8±0.9	8.8±2.8	7.0±1.5	11.8±2.1

Shunt, QP/QS	2.2±0.8	1.6±0.6	1.0±0.1	1.0±0.1

## Conclusions

The shunting ratio in ASD patients decreases during dobutamine stress. This is explained by proportionally larger increase in systemic flow compared to shunting flow. Exercise capacity in patients is related to the capacity to maintain a high systemic cardiac output during stress but not to the degree of shunting, RV function at stress or to LAP or PAP at rest.

## Funding

The Swedish Heart-Lung Foundation.

